# High‐Performance Graphene‐Based Cementitious Composites

**DOI:** 10.1002/advs.201801195

**Published:** 2019-03-07

**Authors:** Małgorzata Krystek, Dawid Pakulski, Violetta Patroniak, Marcin Górski, Leszek Szojda, Artur Ciesielski, Paolo Samorì

**Affiliations:** ^1^ Department of Structural Engineering Faculty of Civil Engineering Silesian University of Technology Akademicka 5 44‐100 Gliwice Poland; ^2^ Université de Strasbourg CNRS, ISIS 8 alleé Gaspard Monge 67000 Strasbourg France; ^3^ Faculty of Chemistry Adam Mickiewicz University Umultowska 89b 61‐614 Poznań Poland; ^4^ Centre for Advanced Technologies Adam Mickiewicz University Umultowska 89c 61‐614 Poznań Poland

**Keywords:** consistency, electrochemically exfoliated graphene, mechanical properties, Portland cement, structural characterization

## Abstract

This study reports on the development of a cementitious composite incorporating electrochemically exfoliated graphene (EEG). This hybrid functional material features significantly enhanced microstructure and mechanical properties, as well as unaffected workability; thus, it outperforms previously reported cementitious composites containing graphene derivatives. The manufacturing of the composite relies on a simple and efficient method that enables the uniform dispersion of EEG within cement matrix in the absence of surfactants. Different from graphene oxide, EEG is found to not agglomerate in cement alkaline environment, thereby not affecting the fluidity of cementitious composites. The addition of 0.05 wt% graphene content to ordinary Portland cement results in an increase up to 79%, 8%, and 9% for the tensile strength, compressive strength, and Young's modulus, respectively. Remarkably, it is found that the addition of EEG promotes the hydration reaction of both alite and belite, thus leading to the formation of a large fraction of 3CaO·2SiO_2_·3H_2_O (C‐S‐H) phase. These findings represent a major step forward toward the practical application of nanomaterials in civil engineering.

## 1 Introduction

Nowadays, with its global consumption reaching 30 Gt per year, concrete is the most consumed synthetic material in the world, and the second only to water as the most consumed material on the global scale.^1^, ^2^ Despite the fact that concrete is presently the most common material applied for building structures, cement composites still possess several significant drawbacks. The major limitation of concrete is its inherent quasi‐brittle nature attributable to the high compressive strength and to the relatively low tensile strength.^2^ These properties result in high vulnerability to cracking; thus, the exposure to the environmental conditions unfavorably affects the durability of concrete structures.^3^ Moreover, the environmental impacts of concrete production cannot be ignored. Cement production is an energy‐intensive process accounting for 7% of the industrial energy consumption and that constitutes 2%–3% of the total global energy use.^1^, ^4^ Even more alarming is the amount of water irreversibly lost in cement matrix. Concrete production makes use of 18% of the global industrial water consumed per year and accounts for 9% of global annual industrial water withdrawals.^5^ Furthermore, since the production of 1 ton of cement corresponds to emission of ≈1 ton of carbon dioxide, cement industry is responsible for 8%–9% of the release of anthropogenic CO_2_ into the atmosphere annually.^1^ Because of these reasons, a key target for concrete technology is to improve the performance of cementitious materials toward reducing the consumption of concrete components.

Typically, various types of reinforcements, including steel rebars, fiber‐reinforced polymer rebars, carbon fibers, glass fibers, steel fibers, or polypropylene fibers, are applied in concrete members to ensure the required moment or shear resistance by controlling the initiation and propagation of cracks.^2^, ^6^ Nevertheless, these kinds of reinforcements do not affect cement hydration products, thus the brittleness and cracking still occur at the nanoscale.^2^ Within this framework, the application of principles of nanotechnology in concrete structures has recently emerged as an attractive solution. The incorporation of nanomaterials, including metal oxide nanoparticles, such as nanotitania, nanoalumina, nanosilica, and nanoiron oxide, as well as nanocarbon additives, namely, carbon nanotubes or graphene (G) derivatives, can offer major improvements in cement materials ranging from enhancement of already existing properties to providing completely new functionalities and capabilities. Nanotechnology innovations for construction industry comprise high strength, durability and sustainability of structural composites, self‐cleaning, antimicrobial, anticorrosion, and air purifying surfaces, as well as sensing devices and self‐sensing members for structural health and safety monitoring.^7^, ^8^

Since the discovery of multi‐walled^9^ and single‐walled^10^ carbon nanotubes (CNTs) in 1990s, CNTs have become the most studied carbon nanomaterial in concrete technology.^11^, ^12^, ^13^, ^14^, ^15^, ^16^, ^17^ However, the production of novel cement nanocomposites involves the perplexing problem of disentanglement and uniform distribution of nanomaterials within cement matrix. Carbon‐based nanoparticles are prone to form agglomerates and bundles in aqueous environment, thereby hindering their uniform dispersion in cement paste.^12^, ^15^, ^18^ Nonuniformly dispersed nanomaterials may be detrimental to the microstructure of cement composite and consequently worsening its mechanical properties. Therefore, the development of an efficient method for obtaining the homogenous dispersion of CNTs within cement matrix turned out to be an extremely challenging task. Examples that have been reported to date involve functionalization^15^ and acid treatment^16^ of CNTs, as well as stirring and ultrasonication of CNTs with various types of surfactants^11^, ^12^, ^14^, ^17^ or solvents.^13^ The addition of CNTs can, in particular, enhance the mechanical properties of cement composites^11^, ^15^, ^16^, ^17^ (see **Table** **1**), refine their pore structure,^11^, ^14^, ^15^, ^16^ and reduce the drying shrinkage.^15^ The strengthening mechanism of CNTs may be attributed to the higher formation of strong 3CaO·2SiO_2_·3H_2_O (C‐S‐H) phase,^14^ as well as to the bridging effect between cracks and pores in composites' structure.^11^, ^16^ Noteworthy, the microstructure and mechanical properties of cementitious composites incorporating CNTs are highly dependent on the degree of dispersion of CNTs: significantly deteriorated performance of processed composites has been reported.^13^

**Table 1:** Enhancement of the mechanical properties of cement‐CNTs and cement–GO composites

	Ref.	CNTs/GO loading	Sample shape and size	Strength increasea)
CNTs	13	0.5 wt%	Rectangular 40 × 40 × 160 mm	Compressive strength: increase of 11% for pristine CNTs, 17% for annealed CNTs, decrease of 86% for carboxy‐group functionalized CNTs
	15	0.1 wt% 0.3 wt%	Cubic 40 × 40 × 40 mm Rectangular 40 × 40 × 160 mm	Compressive strength: 7% Flexural strength: 6%
	16	**0.5 wt%**	Cubic 40 × 40 × 40 mm Rectangular 40 × 40 × 160 mm	**Compressive strength: 19%** Flexural strength: 25%
	17	0.5 wt% **0.1 wt%**	Cubic 40 × 40 × 40 mm Rectangular 40 × 40 × 160 mm	Compressive strength: 11% **Flexural strength: 86%**
	14	0.048 wt.%	Rectangular 20 × 20 × 80 mm	Flexural strength: 25%
	12	0.05 wt%	Dumbbell‐shaped cylinders with a diameter of 2 mm	Compressive strength under dynamic loading: 40%
GO	26	0.04 wt% **0.03 wt%**	Cubic 40 × 40 × 40 mm Rectangular 40 × 40 × 160 mm	Compressive strength: 25% **Flexural strength: 57%**
	32	0.2 wt.%	Cubic 40 × 40 × 40 mm	Compressive strength: 11%
	34	0.022 wt%	Cubic 40 × 40 × 40 mm Rectangular 40 × 40 × 160 mm	Compressive strength: 23% Flexural strength: 25%
	29	0.04 wt%	Cubic 20 × 20 × 20 mm	Compressive strength: 15%
	25	0.05 wt%	Cubic 15 × 15 × 15 mm Rectangular 15 × 15 × 80 mm	Compressive strength: 24% Flexural strength: 49%
	27	**0.08 wt%** 0.04 wt%	Cylindrical 25 × 50 mm Rectangular 15 × 30 × 140 mm	**Compressive strength: 47%** Flexural strength: 14%
	28	**0.04 wt%**	Cylindrical 23.5 × 47 mm	**Tensile splitting strength: 67%**

^a)^ The highest increase of compressive, flexural, and tensile strength for both CNTs and GO has been shown in bold.

In the last decade, graphene, a 2D honeycomb lattice of sp^2^ carbon atoms,^19^, ^20^, ^21^ has attracted the major attention in science and technology as an exciting material holding numerous outstanding properties. In particular, the graphene's unprecedented mechanical properties^22^, ^23^ make it one of the most promising nanomaterials for application in composites,^24^ and in particular for civil engineering applications.

However, the research on the potential application of graphene in cement composites is still at its infancy: as in the case of CNTs, the generation of homogeneous dispersion of graphene within cement mix represents a major problem to be solved. Therefore, cement composites incorporating graphene oxide (GO), i.e., the most easily processable graphene derivative, have been intensively investigated in recent years.^25^, ^26^, ^27^, ^28^, ^29^, ^30^, ^31^, ^32^, ^33^, ^34^ Graphene oxide is highly dispersible in water due to the oxygen functional groups attached on the basal plane and edges of GO sheets,^35^ hence the main approach employed to fabricate cement–GO composites involves simply ultrasonication of GO dispersion in water, prior to mixing with cement.^25^, ^26^, ^27^, ^28^ However, it has been reported that the addition of GO notably increases the yield stress and plastic viscosity of cement paste, thus affecting its fluidity and highly reducing its workability.^25^, ^29^, ^30^ The authors attributed this phenomenon to the reduction of free water in cement mix due to the large surface area of GO nanosheets absorbing more water to wet their surface.^25^ Nonetheless, as revealed by recent studies,^29^, ^31^, ^32^ the incorporation of graphene oxide dispersion into cement leads to the immediate formation of agglomerations and flocculation due to the formation of metal complexes, i.e., chemical cross‐linking of GO nanosheets by calcium ions present in cement matrix. The GO aggregates entrap free water molecules, thereby affecting the rheological properties of cement mixture. In order to provide the appropriate workability of composites and the sufficient dispersion of GO in alkaline cement environment, two main approaches have been proposed. The former relies on the surface modification of GO by mixing and sonication with the addition of polycarboxylate superplasticizer (PC) to prevent agglomeration of GO, by taking advantage of the strong steric hindrance effects of PC to separate GO nanosheets from Ca^2+^ ions.^33^, ^34^ The latter consists in the addition of silica fume in order to separate GO nanosheets from charged ions, in the form of combined aqueous dispersion of GO‐SF^31^ or graphene oxide encapsulated silica fume particles.^29^

The cement–GO composites have emerged as materials with improved mechanical properties (Table 1). Even in the case of composites with lowered workability, where no special treatment for ensuring appropriate GO dispersion within cement matrix was applied,^25^, ^26^, ^27^, ^28^ the addition of GO has greatly enhanced the strength of cement paste. The maximum increase up to 47%, 57%, and 67% has been noted for compressive,^27^ flexural,^26^ and indirect tensile strength,^28^ respectively. However, these results may be explained in terms of the scope of performed tests. Indeed, the fabrication of relatively low volume of cement mix is much less affected by poor fluidity and decreased setting time as in the case of tests on a broader scale. It should be emphasized that although obtaining GO dispersion in water via ultrasonication is a relatively simple method for preparing cement nanocomposite in laboratory conditions, it will suffer from crucial technological and workability problems in industrial scale applications.

Mechanical properties of cementitious composites should be assessed by means of their microstructure and composition of cement paste hydrates. As demonstrated in previous studies, the reinforcing mechanism of GO is attributed to the chemical reaction between GO nanosheets and cement hydration products.^25^, ^30^ Due to a large amount of oxygen functional groups attached on the GO sheets and its high specific surface area, GO promotes the growth of cement hydration crystals by the nucleation effect. The network structure composed of GO nanosheets and hydration products is a result of chemical reaction between —COOH in GO and Ca^2+^ in calcium hydroxide.^26^

While the potential application of graphene oxide in cement composites has been extensively studied in recent years, similar research involving graphene, to date, remained limited. Some attempts have been made to fabricate cement composites with the addition of graphene nanoplatelets.^18^, ^36^, ^37^, ^38^, ^39^ However, similarly as in the case of graphene oxide, the application of polycarboxylate,^36^ methylcellulose,^37^ or naphthalene‐sulfonate based^18^, ^38^, ^39^ surfactants was necessary to ensure the appropriate dispersion of nanomaterial within cement matrix. Interestingly, the cement paste incorporating the aqueous solution of graphene has been also investigated.^40^ This particular composite has emerged as a material with the decreased compressive and flexural strength. The results have indicated that the addition of graphene was detrimental to the microstructure of cement paste and it inhibited the development of cement hydration due to the hydrophobic behavior of graphene and its tendency to form agglomerates in cement matrix.

Here we present an unprecedented cementitious composite incorporating few‐layer thick graphene nanosheets. We have devised a technologically simple yet efficient method for manufacturing a cement–graphene composite based on the use of graphene obtained via electrochemical exfoliation (EE) of graphite foil. Prior to being mixed with cement, the graphene aqueous suspension is dried and wiped through the set of sieves. Contrary to previous reports describing the generation of cementitious materials incorporating graphene derivatives, the preparation of our composite does not require special treatments or the use of surfactant to obtain uniform dispersion of graphene within cement matrix. We first evaluated the dispersion of graphene in cement alkaline environment and its effect on the consistency of cement mortar. Then we investigated the mechanical properties of produced composites to select the most advantageous content of graphene. The 0.05 wt% addition of graphene results in the highest increase of mechanical properties, namely, 79%, 8%, and 9% for tensile strength, compressive strength, and Young's modulus, respectively. Finally, we characterized the microstructure and composition of our mortars. As revealed by detailed characterization, the hydration rate of calcium silicates is remarkably increased in cement–graphene composites. The addition of electrochemically exfoliated graphene (EEG) accelerates the nucleation and growth of C‐S‐H phase, thus resulting in significant improvement of composites' strength. Subsequently, the fluidity of the processed composites remains unaltered compared to reference mortars, indicating that the concrete alkaline environment does not jeopardize the uniform dispersion of graphene within cement matrix. These results open new possibilities for the practical applications of graphene in high‐performance cementitious composites.

## 2 Results and Discussion

### 2.1 Morphology of Electrochemically Exfoliated Graphene

EEG has been prepared under anodic conditions by using a simple electrolytic cell.^41^, ^42^, ^43^, ^44^ In general, the oxidation of graphene sheets is unavoidable during anodic EE, and it depends on both the exfoliation time and the type of employed electrolyte, which in some cases can prevent extensive oxidation.^45^ On the other hand, the amount of the exfoliated material increases with the EE time, which makes the EE process appealing from industrial perspective, as it can be easily upscaled. In particular, an electrolysis process lasting 180 min allows production of ≈200 mg of EEG, whereas classical EE lasting for 10 min results in ≈10 mg of EEG.^44^ Noteworthy, during the electrolysis process, the area of the working electrode (graphite foil) is reduced, determining a variation of the current intensity passing between the electrodes. Interestingly, long‐standing electrolysis in aqueous solution impacts the oxidation degree of the produced material.

**Figure 1:**
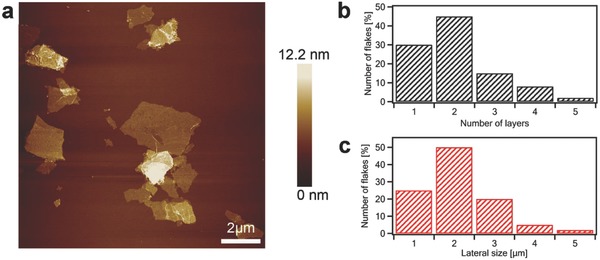
a) AFM topography image of the electrochemically exfoliated graphene flakes deposited on SiO_2_ substrates. b) Lateral size distribution of graphene flakes. c) Histogram of the number of layers per sheet.

Atomic force microscopy (AFM) is employed to gain in‐depth insight into the surface morphology of EEG flakes. Toward this end, EEG flakes were deposited on SiO_2_ substrate by spin coating 200 µL of 1 mg mL^−1^ dispersion in dimethylformamide. **Figure** **1**a displays large single and few‐layer graphene sheets. AFM enables estimation of the number of layers by measuring the height of the deposited flakes from topographical profiles and dividing it by the graphite interlayer distance. The lateral size of EEG sheets was found to vary between 2 and 5 µm, being a typical characteristic of graphene produced via the EE process (Figure 1b). The EEG possesses a high yield (>80%) of one to three‐layer thick graphene flakes, including bilayer graphene (≈45%) as a main product (Figure 1c).

### 2.2 X‐Ray Photoelectron Spectroscopy Analysis of Electrochemically Exfoliated Graphene

**Figure 2:**
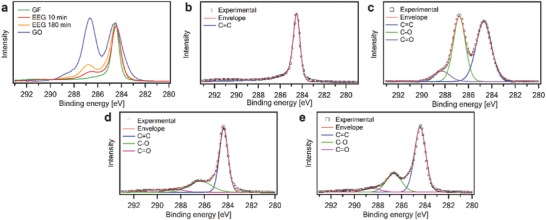
a) Overlapped high‐resolution carbon materials spectra. XPS spectra of b) graphite, c) graphene oxide, and EEG after d) 10 min and e) 180 min of exfoliation, respectively.

The chemical composition of the as prepared EEG flakes was unraveled by X‐ray photoelectron spectroscopy (XPS) and compared with the starting material, i.e., the graphite foil, as well as with commercially available graphene oxide. Such analysis was performed aiming at following the changes in the chemical composition of the material during EE. **Figure** **2** displays a comparison between C1s spectra of the starting material, graphene oxide, and EEG. As previously reported, the high‐resolution C 1s spectrum of the starting material (Figure 2b) displays an asymmetric peak observed at 284.5 eV.^42^ After exfoliation, the C 1s spectra of EEG powder (Figure 2d,e) show the main peak centered at 284.5 eV and the presence of two other components at higher binding energies, which indicates oxidation of the material during EE. The deconvoluted XPS C 1s spectra reveal the presence of oxygen‐containing groups, i.e., epoxide (286.6 eV) and carbonyl (288.3 eV) functional groups (Figure 2a). From ≈10% (for 10 min EEG process) to ≈22% (for 180 min EEG process) of oxygen is present in EEG, attributable to the oxidation of graphene, which is unavoidable during the electrochemical process, given from the XPS survey spectra (see Figure S1 in the Supporting Information). High C/O ratio of about 10 can be achieved after 10 min of EEG process, in contrast to C/O ratio ≈3.5 after 180 min process.

### 2.3 Consistency

We first investigated the dispersion of EEG and GO in alkaline environment and their influence on the consistency of cement mortar. The solution present in concrete pores is highly alkaline due to the presence of cations, including Ca^2+^, Na^+^, and K^+^ and anions such as OH^−^ and (SO_4_)^2−^. Ca^2+^ and OH^−^ ions originate from calcium hydroxide, Na^+^ and K^+^ ions exist in sodium and potassium oxides present in cement, while sulfate ions can originate from gypsum, mixing water, or aggregate.^46^, ^47^ Several attempts have been made to evaluate the performance of GO dispersion in concrete pore solution, namely, by using separate solutions of Ca(OH)_2_, NaOH, KOH, NaCl, CaCl_2_, and NH_3_ x H_2_O^31^, ^32^, ^34^, ^48^ or by simulating mixed pore solution of Ca(OH)_2_, NaOH, and KOH.^48^ However, GO dispersion proved to remain stable in the presence of Na^+^ and K^+^ ions in the solution with pH value below 13.^48^ Placing these findings into cement perspective with pH value of fresh Portland cement paste of ≈12.5,^46^ sodium and potassium ions should not hinder the spreading of graphene derivatives within cement matrix. Conversely, even low quantities of calcium ions may yield the immediate formation of GO agglomerates.^31^, ^32^, ^48^ Therefore, we aimed to assess the stability of EEG and GO dispersion in cement alkaline environment in the presence of Ca^2+^ ions. Toward this end, we added 100 µL of saturated Ca(OH)_2_ solution to 3.0 mL of aqueous dispersion of EEG or GO dispersion with concentration of 0.07 mg mL^−1^. Indeed, the addition of Ca(OH)_2_ results in rapid and notable flocculation of GO, conversely not affecting graphene dispersion (Figure S2, Supporting Information).

**Figure 3:**
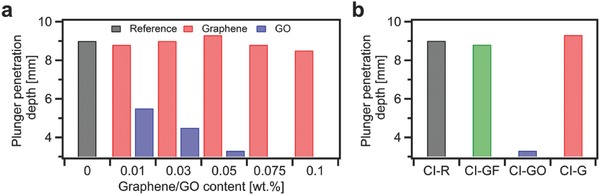
Consistency measurements for a) CEM I composites incorporating graphene and graphene oxide, b) CI‐R, CI‐GF‐0.05, CI‐GO‐0.05, and CI‐G‐0.05 samples.

We then attempted to manufacture several cement mortars made out of Portland cement Type I with various loadings of EEG and GO ranging from 0 wt% to 0.1 wt%. Noteworthy, the water‐to‐cement and sand‐to‐cement ratios remained 0.5 and 3.0, respectively. **Figure** **3**a plots the results of plunger penetration test performed immediately after manufacturing CEM I composites. The consistency measurements confirm our previous observations. Clearly, the addition of GO leads to the immediate reduction of fluidity, whereas the consistency of cement–graphene composites remains relatively unaltered compared to reference mortars. Therefore, our attempts to produce cement mortars with GO loading above 0.05 wt% failed due to the insufficient workability of the composites.

Moreover, in order to provide extensive benchmarking for samples with EEG, we have produced additional CEM I mortar incorporating 0.05 wt% of graphite flakes (GFs). However, as in the case of EEG, we have reported negligible effect of the graphite flakes addition on the consistency of cement mortar (see Figure 3b).

Simultaneously, we decided to evaluate another type of cement, i.e., Portland cement Type II with granulated blast furnace slag. CEM II mortars with EEG and GO were produced as it was described for CEM I. In this case we note that, interestingly, the consistency of mortars is less affected by GO addition than it occurs in CEM I composites (Figure S3, Supporting Information). Given a similar amount of water required to obtain the standard consistency for both types of cement (see Table S1 in the Supporting Information), as well as the same depth of plunger penetration noted for reference samples in consistency test, this phenomenon should be explained in terms of chemical composition of CEM I and CEM II (Table S2, Supporting Information). CEM II modified with granulated blast furnace slag contains less CaO than CEM I. This fact, combined with our consistency measurements, provides evidence that chemical cross‐linking of GO by calcium ions may account for the reduction of cement mortar fluidity, as revealed by previous studies.^29^, ^31^, ^32^

### 2.4 Mechanical Properties

**Figure 4:**
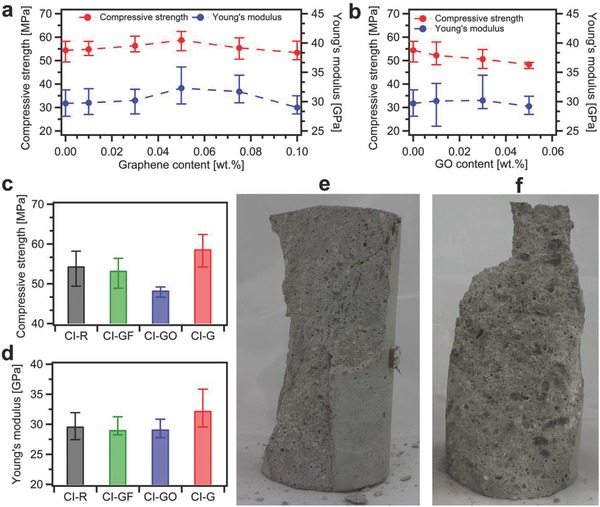
Results of compression tests of CEM I mortars at the age of 28 d. a,b) Compressive strength and Young's modulus values for composites incorporating a) graphene and b) graphene oxide. c) Compressive strength and d) Young's modulus values for CI‐R, CI‐GF‐0.05, CI‐GO‐0.05, and CI‐G‐0.05 samples. e) CI‐G‐0.05 sample after compression test revealing a characteristic cone shape of failure. f) CI‐GO‐0.05 sample after compression test with visible GO agglomerates and large pores.

We then performed compressive and tensile strength tests of all produced mortars at the age of 28 d. In order to obtain the most accurate results not affected by the end restraint effect, we have investigated mechanical properties on cylindrical samples with the height‐to‐diameter ratio of 2 (Figure S4, Supporting Information).^46^ Indeed, a characteristic cone shape of failure of compressed samples (**Figure** **4**e) reveals that the restraining effect of the plates of strength tester is largely eliminated and uniaxial compression occurred in the middle of tested samples.

Figure 4a,b plots the compressive strength of CEM I composites incorporating EEG and GO, respectively. We have found that the samples with 0.05 wt% loading of graphene exhibit the slight increase of compressive strength up to 8%. Simultaneously, the Young's modulus values, determined as a secant value between zero stresses and stresses equal to 40% of compressive strength, show the same trend with the increase of 9% for CI‐G‐0.05 samples.

On the other hand, with the introduction of 0.05 wt% of GO, the compressive strength is considerably decreased by 11% (Figure 4b). This finding can be ascribed, to a great extent, to poor workability and thus insufficient compaction and disorder of mortar components distribution. Moreover, compression tests on CI‐GO‐0.05 samples revealed the damaged surface with highly visible GO agglomerates and large pores (Figure 4f), a further indication on the inhomogeneous distribution of mortar components. In addition, the effect of graphite flakes on the compressive strength and Young's modulus turned out to be negligible (Figure 4c,d).

**Figure 5:**
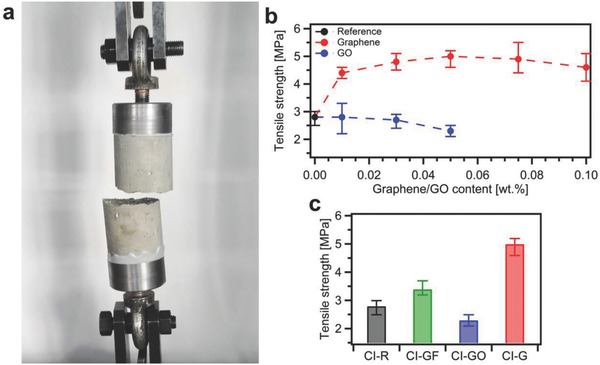
Tensile strength tests of CEM I mortars at the age of 28 d. a) CI‐R sample after uniaxial tension test. b) Tensile strength for composites incorporating graphene and graphene oxide. c) Tensile strength for CI‐R, CI‐GF‐0.05, CI‐GO‐0.05, and CI‐G‐0.05 samples.

Furthermore, since it is of crucial importance to improve concrete performance in tension, we have then determined the tensile strength of produced mortars by means of a direct tension test (**Figure** **5**a). Here we note the significant improvement of uniaxial tensile strength for all composites incorporating EEG, even at the lowest graphene content, i.e., 0.01 wt% (Figure 5b). Also in this case, as for the compressive strength and Young's modulus, 0.05 wt% loading of EEG proved to be the most beneficial. The tensile strength of CI‐G‐0.05 specimens is remarkably increased by 79%.

Significantly, the remarkable tensile strength values we achieved cannot be directly compared with those reported in the literature,^28^ since the studies are different in many aspects. In particular, the strength tests may be affected by specimen size and type, whether the sample is cubic or cylindrical, rate of loading, water‐to‐cement ratio, or use of any additives. However, as aforementioned, for composites incorporating GO, the maximum increase up to 67% of indirect tensile strength has been noted in previous studies.^28^ Nevertheless, it should be highlighted that these tensile splitting tests were conducted on cylindrical samples of cement paste with dimensions of 23.5 × 47 mm, indicating the strong influence of size effect on the reported results.

Clearly, since large pores have been monitored in cement–GO composites (Figure 4f), the tensile strength cannot remain unaltered in that case. Indeed, the GO addition results in notable decrease of tensile strength of 18% for CI‐GO‐0.05 samples. Interestingly, although we achieve comparable values of the compressive strength in reference samples and samples with graphite flakes, the addition of graphite improves the tensile strength by 21% (Figure 5c). These results, together with the compressive strength results, may suggest that graphite flakes do not influence the microstructure of cement paste acting similarly to dispersed fiber reinforcement.

Simultaneously, CEM II composites incorporating EEG demonstrate slightly enhanced tensile strength (Figure S5c, Supporting Information) and, surprisingly, decreased compressive strength (Figure S5a, Supporting Information). The effect of GO addition on CEM II mortars strength and Young's modulus is, however, comparable with that noted for CEM I (Figure S5b, Supporting Information).

### 2.5 Microstructure and Composition

In order to understand the origin of the improvement of mechanical properties, as well as the underlaying strengthening mechanism of EEG, we investigated the microstructure and composition of produced cement mortars using various characterization techniques, including thermogravimetric analysis (TGA), X‐ray powder diffraction (XRD), Fourier transform infrared spectroscopy (FTIR), scanning electron microscopy (SEM), and Brunauer–Emmett–Teller (BET) adsorption–desorption analysis.

Fundamentally, mechanical properties of cementitious composites originate from microstructure and composition of cement paste hydrates, in particular, calcium hydroxide, Ca(OH)_2_, and calcium silicate hydrate, C‐S‐H phase. C‐S‐H amorphous phase, accounting for ≈50%–70% of cement structural components, is considered as the strongest phase in hardened cement paste and plays the major role in developing macroproperties of cementitious composites. Ca(OH)_2_ with its hexagonal plate‐shaped crystals, being a considerably weaker phase, is responsible for providing high alkalinity of concrete environment, thereby protecting steel reinforcement against corrosion. However, high amount of calcium hydroxide may contribute to leaching, carbonation, alkali aggregate reaction, or sulfate attack, thus deteriorating the durability of concrete structures.^49^

Since the determination of C‐S‐H and Ca(OH)_2_ content is of significant importance in characterizing cementitious materials, we first performed thermogravimetric analysis to probe Ca(OH)_2_ amount in all produced mortars at the age of 28 d. TGA has been shown to be a powerful tool for estimating portlandite content in cement composites, considering the specimens weight losses in the dehydroxylation (≈400–550 °C) and decarbonation (≈600–800 °C) regions.^26^, ^32^, ^50^ In this case, on the basis of thermogravimetric derivative (DTG) curve (Figure S6, Supporting Information), the calcium hydroxide content was determined according to the following equation

(1) $$\mathrm{CH}={\mathrm{CH}}_{\mathrm{ldh}}+{\mathrm{CH}}_{\mathrm{ldc}}=\frac{\mathrm{ldh}*m_{\mathrm{CH}}}{m_{H2O}}+\frac{\mathrm{ldc}*m_{\mathrm{CH}}}{m_{\mathrm{CO}2}}$$

where ldh represents the percentage of mass loss in the dehydroxylation region (400–530 °C); ldc is the percentage of mass loss in the decarbonation region (600–750 °C); *m*_H2O_, *m*_CH_, and *m*_CO2_ correspond to the molecular mass of H_2_O (18 g mol^−1^), Ca(OH)_2_ (74 g mol^−1^), and CO_2_ (44 g mol^−1^), respectively.

**Figure 6:**
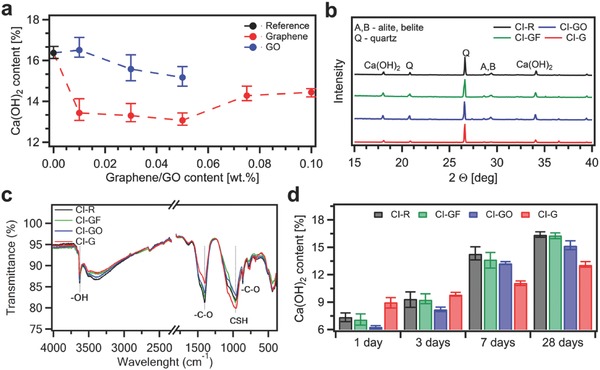
Structural characterization of CEM I composites. a) Ca(OH)_2_ content at the age of 28 d in cement mortars incorporating graphene and graphene oxide. b) XRD patterns and c) FTIR spectra for CI‐R, CI‐GF‐0.05, CI‐GO‐0.05, and CI‐G‐0.05 samples at the age of 28 d. d) Ca(OH)_2_ content at the age of 1, 3, 7, and 28 d in CI‐R, CI‐GF‐0.05, CI‐GO‐0.05, and CI‐G‐0.05 samples.

**Figure** **6**a shows the portlandite content in 28 d CEM I composites incorporating EEG and GO. We emphasize that the plot reveals a significant correlation with the results of mechanical properties tests. On the one hand, after the addition of EEG, the Ca(OH)_2_ content is drastically reduced, achieving the lowest value of 13.07% for CI‐G‐0.05 samples, while an average value for reference samples is 16.37%. It should be pointed out that the lowest portlandite content occurred in samples exhibiting the highest tensile strength. On the other hand, we also report the slight decrease of Ca(OH)_2_ amount in GO composites, i.e., from 16.37% to 15.17% for CI‐GO‐0.05 samples. Since these specimens possess the lowest compressive and tensile strength, these results indicate the opposite trend in Ca(OH)_2_ amount compared to graphene composites.

To fully explain our observations, it is particular important to attain a deep understanding on the origin of Ca(OH)_2_ presence in hardening cement matrix. First, at initial contact with water, cement grains start to dissolve, thus releasing, among others, calcium and hydroxide ions from their surface. At the end of dormant period, when the supersaturation point of Ca^2+^ ions is reached, Ca(OH)_2_ starts to precipitate from the saturated solution. Second, Ca(OH)_2_ is produced as a result of hydration reactions (Equation (2) and (3)) of two main cement compounds, namely, tricalcium silicate 3CaO·SiO_2_ (alite) and dicalcium silicate 2CaO·SiO_2_ (belite)^46^, ^49^

(2) $$2\left[3\mathrm{CaO}\cdot {\mathrm{SiO}}_{2}\right]+6H_{2}O\to 3\mathrm{CaO}\cdot 2{\mathrm{SiO}}_{2}\cdot 3H_{2}O+3\mathrm{Ca}{\left(\mathrm{OH}\right)}_{2}$$

(3) $$2\left[2\mathrm{CaO}\cdot {\mathrm{SiO}}_{2}\right]+4H_{2}O\to 3\mathrm{CaO}\cdot 2{\mathrm{SiO}}_{2}\cdot 3H_{2}O+\mathrm{Ca}{\left(\mathrm{OH}\right)}_{2}$$

Noteworthy, although these two reactions result in the same hydration products, they differ considerably in hydration rate, heat, and the amount of produced Ca(OH)_2_. Alite with its fast reaction and high release of heat is responsible for early hydration, thus contributing mainly to early‐age strength of concrete, whereas the reaction of belite being markedly slower releases less heat and contributes to long‐term strength.^46^, ^49^

Since the formation of portlandite is a highly complex process extensively varying at the different stages of ongoing cement hydration, we have then performed XRD and FTIR analysis on samples cured for 28 d, in order to gain further insight onto the course of cement hydration reactions and their products. We have focused our attention on four representative samples made out of CEM I, namely, CI‐R, CI‐G‐0.05, CI‐GF‐0.05, and CI‐GO‐0.05.

Figure 6b,c portrays the XRD patterns and FTIR spectra of selected CEM I samples (see also Figure S7 in the Supporting Information). Since the amount of sand was kept constant in all mixes, XRD patterns were scaled up by the intensity of major Quartz peak at position of 2*Θ* = 26.6°.^51^ The peaks for Ca(OH)_2_ appear at 18.0° and 34.1°.^51^, ^52^ Indeed, XRD results confirm visibly the decrease of Ca(OH)_2_ amount in CI‐G‐0.05 samples as revealed previously by TGA. Basically, as C‐S‐H gel is an amorphous phase, it cannot be detected during XRD analysis. However, the hydration degree may be estimated on the basis of remaining peaks of alite and belite at the positions of 28.7° and 29.4°.^51^ As observed for CI‐G‐0.05 sample, the peaks of alite and belite disappeared, indicating that EEG has promoted the hydration reactions, thus leading to a higher degree of hydration compared to plain cement mortar. However, no significant changes for GO samples were detected by XRD.

In the case of FTIR, the peaks attributed to —OH of Ca(OH)_2_ at 3650 cm^−1^,^26^ as well as —C—O of CaCO_3_ at 874 and 1414 cm^−1^,^52^ can be observed. Here we note that FTIR spectra are in line with TGA and XRD studies demonstrating lower content of portlandite in samples incorporating EEG and GO. Moreover, the intense band at 955 cm^−1^ represents the stretching vibration of Si—O bonds of C‐S‐H gel revealing, in particular, higher formation of C‐S‐H phase in cement mortar reinforced with graphene and, simultaneously, restrained C‐S‐H growth in cement–GO composites.

Interestingly, the evaluation of the specific surface area of cementitious composites has been shown being a powerful tool for assessing the C‐S‐H phase development, which is strongly associated with the degree of cement hydration.^25^ Because of this reason, we have characterized the porosity of CI‐R, CI‐G‐0.05, and CI‐G‐0.05 specimens by N_2_ adsorption–desorption isotherms measurement at 77 K (Figure S8a, Supporting Information). CI‐R composite displays a specific surface area of ≈9 m^2^ g^−1^, while the specific surface area of CI‐GO and CI‐G is calculated with the BET model as 17 and 29 m^2^ g^−1^, respectively. Significantly, the results indicate the increased development of the highly porous phase, i.e., the C‐S‐H gel, resulting from the addition of EEG. Moreover, the average pore diameter calculated with the Barrett–Joyner–Halenda model for CI‐R, CI‐GO, and CI‐G amounts to ≈3.9 nm (Figure S8b, Supporting Information).

We have then extended our studies to TGA measurements of samples cured for 1, 3, and 7 d, allowing us to follow the evolution of Ca(OH)_2_ formation at different stages of cement hydration (Figure 6d). In this regard we note that, initially, EEG promotes the nucleation of calcium hydroxide from saturated solution. On the other hand, when the hydration reactions of alite and belite are in progress, the addition of graphene effects in lower production of Ca(OH)_2_. These findings, combined with XRD and FTIR results, suggest that graphene promotes the hydration of calcium silicates, thus resulting in lower amount of alite and belite remaining in hardened cement paste. Moreover, EEG appears to modify the molar ratio of reaction products (see Equation (2) and (3)), hence leading to much more intense formation of strong C‐S‐H phase and, simultaneously, highly reduced amount of calcium hydroxide.

In the case of GO, Figure 6d shows that at the initial stage of hydration, the precipitation of calcium hydroxide is severely restrained by GO addition, a consequence of entrapping free water molecules by GO agglomerates. Moreover, as revealed by mechanical properties tests, as well as FTIR analysis, the development of hydration is slightly inhibited by GO addition due to poor workability, and thus the inadequate compaction and distribution of mortar components. Thereby, our results highlight the crucial importance of proper fluidity of cement mix while manufacturing cementitious composites on a larger scale.

Significantly, we also observed that no major differences occurred in the diffraction patterns and FTIR spectra of reference samples and samples with graphite flakes, pointing toward similar mineralogical compositions of both materials. This provides unambiguous proof that the addition of graphite flakes, in contrast to EEG, does not affect the microstructure and hydration products of cement composites, acting only as dispersed reinforcement.

**Figure 7:**
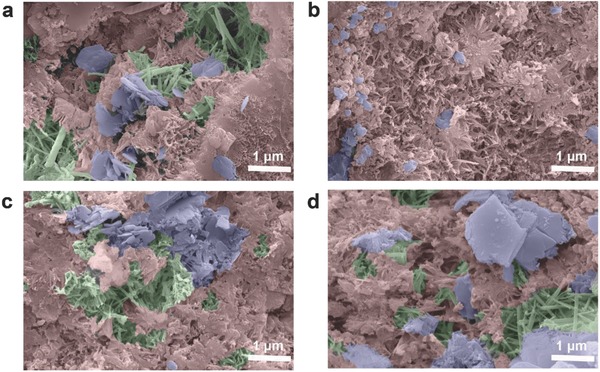
SEM images for a) CI‐R, b) CI‐G‐0.05, c) CI‐GO‐0.05, and d) CI‐GF‐0.05 samples at the age of 28 d. Red, green, and violet colors represent C‐S‐H phase, ettringite, and calcium hydroxide crystals, respectively. Raw SEM images have been shown in the Supporting Information.

Our observations are further confirmed by SEM images (**Figure** **7** and Figure S9, Supporting Information). The image of CI‐G‐0.05 specimen shows, indeed, regular and compact microstructure composed mainly of C‐S‐H phase. Moreover, agglomerations of needle‐like crystals, i.e., ettringite, and plate‐like crystals of Ca(OH)_2_ can be easily observed in plain, GO, and graphite composites, while they do not occur in graphene mortar. The visible densification of microstructure provides a rational explanation of the remarkable performance of cement‐EEG mortars in mechanical properties tests.

In addition to our findings in terms of Portland cement Type I, structural characterization of CEM II composites was performed using the same techniques previously described. The Ca(OH)_2_ content estimated using TGA turned out to be reduced marginally in samples incorporating EEG (Figure S10a, Supporting Information). As in CEM I composites, the lowest value of Ca(OH)_2_ occurred in samples with the highest tensile strength. Nevertheless, the value changes only from 11.21% in reference samples to 10.55% in CII‐G‐0.05 specimens. Interestingly, the addition of GO results in intense formation of calcium hydroxide, achieving a value as high as 13.8% for CII‐GO‐0.05 samples. These observations are also supported by XRD (Figure S10b, Supporting Information) and FTIR (Figure S10c, Supporting Information) analysis. Moreover, XRD patterns reveal a slight decrease in alite and belite amount for samples with graphene, while FTIR spectra show a similar trend in C‐S‐H phase formation as in CEM I.

Particular attention should be paid to Ca(OH)_2_ content after 1, 3, and 7 d of cement hydration. In Portland cement with the addition of granulated blast furnace slag, Portland cement components start to hydrate first. At this initial stage, the hydration of CEM II strongly resembles that of ordinary Portland cement.^46^ Indeed, this phenomenon is highly visible in Figure S10d (Supporting Information). However, after 3 d notable decrease in Ca(OH)_2_ content for reference sample is noted, a fingerprint of the presence of granulated blast furnace slag. Basically, when Portland clinker reactions are in progress, granulated blast furnace slag is then activated by alkalis and starts to react with calcium hydroxide released by Portland cement. As a consequence, additional amount of C‐S‐H phase is formed.^46^ Our results suggest that although graphene does promote hydration of Portland cement resulting in higher C‐S‐H nucleation, it constrains, to a certain extent, the activation of slag. Because of these reasons, the influence of EEG on mechanical properties of CEM II mortars turned out to be marginal. Moreover, GO proved to be detrimental for both Portland clinker hydration and slag reaction with calcium hydroxide.

## 3 Conclusion

In summary, we have demonstrated a novel cementitious nanocomposite incorporating graphene that features remarkably enhanced mechanical properties and microstructure. We first established a technologically simple and efficient method for producing a cement–graphene composite, which can be easily employed in industrial scale applications. At the core of our approach is graphene obtained by electrochemical exfoliation of graphite, dried and wiped through the set of sieves, prior to mixing with cement. The preparation method described allows manufacturing a nanocomposite without the use of surfactants or any special treatment to obtain the homogenous dispersion of graphene within cement matrix. In this regard, our composite significantly outperforms previously reported composites with graphene derivatives. Electrochemically exfoliated graphene proved not to aggregate in alkaline environment and thereby does not reduce the fluidity and workability of cement mortars. We then investigated the mechanical properties of produced composites. Remarkably, the addition of 0.05 wt% of graphene to ordinary Portland cement results in significant enhancement of tensile strength up to 79% and, simultaneously, slight increase of compressive strength and Young's modulus by 8% and 9%, respectively. As revealed by further structural characterization, graphene promotes the hydration reactions of calcium silicates, thus resulting in much more intense formation of C‐S‐H phase, as well as regular and compact microstructure. Overall, such findings provide unambiguous evidence that our composite can boost the practical application of graphene in concrete technology. Moreover, the improved performance of cementitious composites incorporating graphene, in particular the significantly enhanced tensile strength, will allow designing lighter concrete structures with extended durability. Therefore, the consumption of concrete components may be reduced, thus mitigating the environmentally harmful impacts of concrete production.

## 4 Experimental Section

*Materials*: Cement, sand, distilled water, graphene, graphene oxide, and graphite flakes were used in this study to fabricate cement mortar. In particular, two types of cement were employed, which were provided by Gorazdze Cement S.A.: CEM I 42.5R (Portland cement Type I) and CEM II/B‐S 32.5R‐NA (Portland cement Type II with granulated blast furnace slag). The chemical composition of the cement is presented in Table S2 (Supporting Information). The mechanical and other physical properties of the cement are reported in Table S1 (Supporting Information). The results of sand sieve analysis are plotted in Figure S11 (Supporting Information). Dispersion of graphene oxide sheets with concentration of 4 mg mL^−1^ was purchased from Graphenea Inc. Graphite flakes were purchased from Sigma‐Aldrich. Graphene was produced by electrochemical exfoliation of graphite foil (Alfa Aesar, 0.5 mm thick) using a platinum wire (GoodFellow, diameter of 0.5 mm) and aqueous solution of ammonium sulfate (NH_4_)_2_SO_4_ (Sigma‐Aldrich). To stop cement hydration, acetone (Carlo Erba Reagents) was used in a process of solvent replacement and liquid nitrogen in a process of freeze‐drying. Dispersions of graphene and graphene oxide in alkaline environment were investigated by using saturated aqueous solution of calcium hydroxide Ca(OH)_2_ (Sigma‐Aldrich).

*Electrochemical Exfoliation of Graphite Foil*: Graphite foil cut into pieces of 2.5 cm x 6 cm was used as anode and platinum wire was used as cathode in an electrolytic cell. Both elements of the electrolytic cell were immersed in aqueous solution of (NH_4_)_2_SO_4_ with concentration of 0.1 m. A starting current of 0.4 A generated by the applied voltage of 15 V (ISO‐TECH IPS‐603 DC power supply) prompted the exfoliation of graphite foil. The exfoliation was conducted for ≈3 h, i.e., until the graphite foil was completely exfoliated. The resulting material was then rinsed several times and filtrated via polytetrafluoroethylene (PTFE) membranes (pores diameter of 5 µm). Finally, collected materials were dried at 60 °C for at least 48 h.

*Cement Mortar Preparation*: Cement mortar was prepared by mixing cement, sand, water, and graphene/graphene oxide/graphite flakes. The water‐to‐cement ratio was kept at 0.5. The sand‐to‐cement ratio was also kept constant in all mixes and it was equal to 3.0. A plain mortar (labeled as R), five mixtures with EEG (labeled as G) and three mixtures containing GO were prepared for each type of cement (CI or CII). Graphene was incorporated in the cement mortar at five different percentages, namely, 0.01 wt%, 0.03 wt%, 0.05 wt%, 0.075 wt%, and 0.1 wt% (dosage by weight of cement). The content of graphene oxide was 0.01 wt%, 0.03 wt%, and 0.05 wt%. Cement mortar with a higher dosage of graphene oxide was not produced due to the reduction of fluidity and setting time, thus resulting in poor workability. Additionally, a reference mixture with 0.05 wt% dosage of GFs was fabricated using CEM I.

For cement mortar containing graphene, dried graphene was wiped through the set of five sieves with the finest mesh sieve size being 250 µm. Graphene and cement were stirred sufficiently at low speed (≈140 rpm) using a hand mixer to obtain a homogenous dry mixture. Mixing procedure from PN‐EN 196‐1:2005 was then adopted. Water was added to cement with graphene and the mixer was immediately started at low speed for 30 s. After that, sand was steadily added during the next 30 s and the mixer was switched to high speed (≈285 rpm) for additional 30 s. Afterward, the mixer was stopped to remove all the mortar adhering the walls of the bowl and then mixing was continued at high speed for 60 s. For samples with graphite, graphite flakes were also added to cement prior to pouring water, whereas for composites with graphene oxide, the GO dispersion was added to cement simultaneously with water. All resulting cement composites were placed into steel cylindrical molds with the diameter of 60 mm and the height of 120 mm. Cement mortar was placed in the molds in a few layers and each layer was subjected to vibration on a vibration table for 1 min to ensure the compaction of the composite. All specimens were immediately covered by polyethylene foil to prevent loss of water. After 24 h, the hardened cement mortar samples were demolded and continued to be cured in water at 20 °C. All cylindrical samples were dried in the air for 24 h before performing mechanical tests.

*Characterization and Measurements*: In order to investigate the dispersion of graphene and graphene oxide in alkaline environment, 3.0 mL of graphene and graphene oxide aqueous dispersions with concentration of 0.07 mg mL^−1^ were first prepared. Then 100 µL of saturated aqueous Ca(OH)_2_ solution was introduced into both dispersions.

The consistency of fresh mortars was determined according to PN‐EN 1015‐4 by the plunger penetration method. The sample was placed in the vessel in two layers and each layer was subjected to vibration on a vibration table. The vessel was then put on the base plate under the plunger. The plunger was allowed to freely fall from the height of 100 mm above the vessel. The depth of the plunger penetration was determined as a consistency measure. Two measurements were performed for each cement mortar and the average was taken.

Mechanical properties tests were conducted on cylindrical samples (Figure S4, Supporting Information) at the age of 28 d. The tensile strength was determined using ZD 10/90 tensile strength tester (Heckert) at a loading rate of 1.0 kN s^−1^. The compressive strength tests were performed using MEGA 3‐3000‐100 compressive strength tester (Form+Test Prüfsysteme) at a loading rate of 0.5 kN s^−1^. Five samples of cement mortar were prepared for each test and the average was taken. To obtain the compression stress–strain curve, linear polyester strain gauges with gauge factor of 2.13 (PFL‐30‐11, Tokyo Sokki) were employed. Two pairs of strain gauges were attached to the two sides of the sample to measure axial and transverse strain. On the basis of the strain gauges measurements, the modulus of elasticity was calculated. According to PN‐EN 1992‐1‐1:2008 the Young's modulus was determined as a secant value between zero stresses and stresses equal to 40% of the average compressive strength.

Inhibiting the cement hydration is essential to prepare cement composites' samples for microstructure and composition characterization by means of TGA, SEM, XRD, FTIR, or BET analyses. Two different methods for stopping the cement hydration in order to obtain the most satisfactory and precise results were employed: freeze‐drying to effectively preserve the composition of cement mortar for TGA, XRD, and FTIR analyses and solvent replacement method to avoid damage of pores and alternations to the cement mortar microstructure for SEM and BET.^53^, ^54^ After mechanical tests, samples were crushed into small pieces of 3–5 mm. The cement mortar pieces intended for solvent replacement method were immediately soaked in acetone for 48 h and then oven‐dried at 40 °C for 48 h. Simultaneously, the remaining mortar pieces were immersed in liquid nitrogen for 10 min and thereafter placed into VirTis BenchTop Pro (SP Scientific) freeze dryer at −82.5 °C and 26 Pa for 72 h. All specimens were subsequently stored in a desiccator until testing.

The microstructure of the fracture surface of hardened cement mortar was investigated by SEM using FEI Dual Beam 235 with the accelerating voltage of 5 keV incident beam energy. The pieces of crushed cement composites after the solvent replacement process were glued to a support with conductive carbon adhesive and the fracture surface was sputter‐coated with a thin layer of gold prior to SEM imaging.

For TGA, XRD, and FTIR analyses, the crushed pieces of cement composites were, initially, grounded completely into fine powder and filtered through a 250 µm sieve to remove coarse grains of sand.

TGA was performed using Mettler Toledo TGA/DSC 2 sensor using alumina crucibles. Three samples of ≈15 mg of each cement mortar were, first, kept isothermally at 30 °C for 30 min and then heated from 30 to 1000 °C at a heating rate of 10 °C min^−1^. All experiments were performed under air atmosphere.

XRD was carried out on Bruker ASX D8 Advanced with Cu anode with Kα radiation (λ = 1.5418 Å). Diffraction patterns were collected at room temperature in the scattered angular range between 6° and 60°. An angular resolution and a typical counting time per each step were of 0.02° and 10 s, respectively.

FTIR spectra were obtained within the mid‐IR range (400–4000 cm^−1^) by using a Perkin Elmer Spectrometer (Spectrum Two) equipped with ATR Diamond.

XPS analyses were performed on a photoelectron spectrometer with a basic chamber pressure of −10^−9^ mbar and an Al anode as the X‐ray source (X‐ray radiation of 1486 eV). Spot size was of 400 µm. Pass energies of 200.00 eV for wide energy scans and 10.00–20.00 eV for scans were used.

The morphology of electrochemically exfoliated graphene was investigated using a Veeco Dimension 3100 AFM with a Nanoscope IV control unit under ambient condition. Topographic imaging was carried out in tapping mode with the use of antimony (n) doped silicon cantilever.

The specific surface area was measured using a Micromeritics ASAP 2050 surface area and porosity analyzer. Prior to the BET measurements, the samples were outgassed for 10 h at 100 °C. Adsorption isotherms were calculated for nitrogen adsorption at 77 K and pressures up to 1 bar.

## Conflict of Interest

The authors declare no conflict of interest.

## Supporting information

Supplementary
