# Supplementary material for: High‐Performance Graphene‐Based Cementitious Composites
Source: Adv Sci (Weinh). 2019 Mar 7;6(9):1801195. doi: 10.1002/advs.201801195 (PMC6498302; doi:10.1002/advs.201801195)
Supplement: Supplementary file 1 — Supplementary [file ADVS-6-1801195-s001.pdf]

Copyright WILEY-VCH Verlag GmbH & Co. KGaA, 69469 Weinheim, Germany, 2019.

ADVANCED SCIENCE Open Access

Supporting Information

for *Adv. Sci.,* DOI: 10.1002/advs.201801195

High-Performance Graphene-Based Cementitious Composites

Małgorzata Krystek, Dawid Pakulski, Violetta Patroniak, Marcin Górski, Leszek Szojda, Artur Ciesielski,* and Paolo Samorì**

Copyright WILEY-VCH Verlag GmbH & Co. KGaA, 69469 Weinheim, Germany, 2016.

Supporting Information

High Performance Graphene-Based Cementitious Composites

Małgorzata Krystek, Dawid Pakulski, Violetta Patroniak, Marcin Górski, Leszek Szojda*, Artur Ciesielski* and Paolo Samorì*

Table S1. Mechanical and other physical properties of CEM I 42.5R and CEM II/B-S 32.5R-NA cement. [a), b)]

	Compressive strength after 2 days [MPa]	Compressive strength after 28 days [MPa]	Start of setting time [min]	Water for standard consistency [%]	Stability of volume [mm]	Specific surface area [cm^2/g]
CEM I 42.5R	28.5	57.6	198	28.3	0.4	3812
CEM II/B-S 32.5R-NA	17.7	48.9	226	28.1	0.3	3755

[a)] Data obtained from Gorazdze Cement S.A.; [b)] All presented values are average values.

Table S2. Chemical composition of CEM I 42.5R and CEM II/B-S 32.5R-NA cement.[a)]

	SiO_2	Al_2O_3	Fe_2O_3	CaO	MgO	SO_3	Na_2O	K_2O	Cl	LOI[b)]	RI[c)]
					[wt.%]						
CEM I 42.5R	19.91	5.14	2.44	63.49	1.72	2.76	0.18	0.66	0.07	3.00	0.76
CEM II/B-S 32.5R-NA	25.06	5.83	1.91	57.47	3.03	2.56	0.26	0.64	0.07	2.58	0.60

[a)] Data obtained from Gorazdze Cement S.A.; [b)] LOI and [c)] RI are the abbreviation of loss on ignition and insoluble residue.

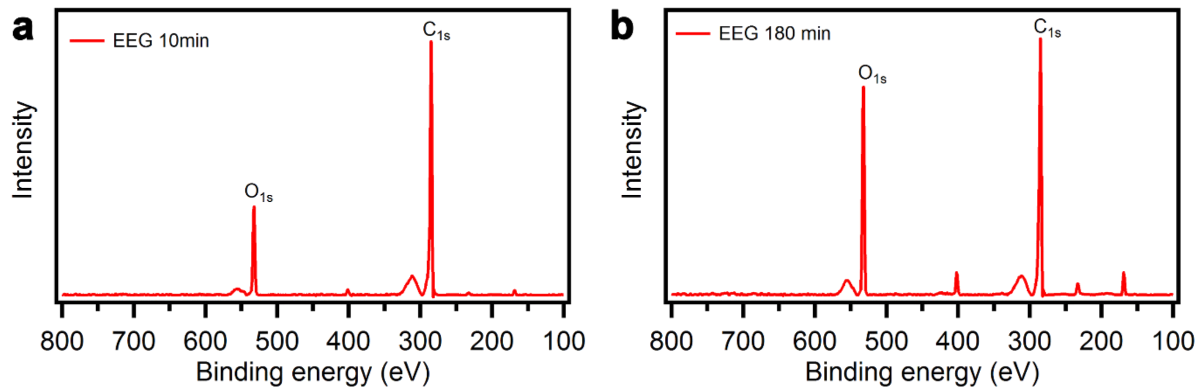

Figure S1. X-ray Photoelectron Spectroscopy (XPS) characterization of electrochemically exfoliated graphene after (a) 10 min and (b) 180 min of exfoliation.

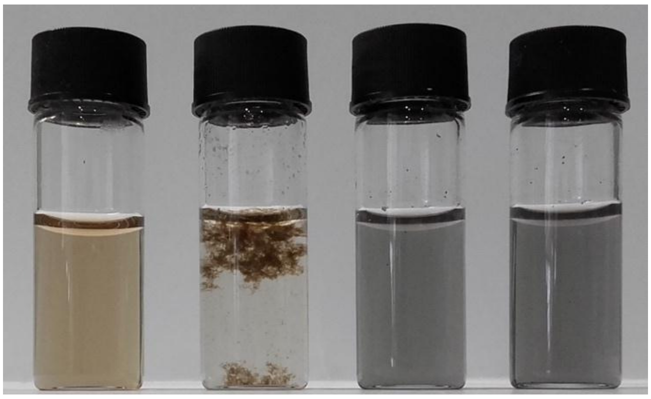

Figure S2. Stability of graphene and GO dispersion in the presence of $Ca(OH)_2$ (from left: aqueous dispersion of GO, aqueous dispersion of GO immediately after adding saturated $Ca(OH)_2$ solution, aqueous solution of graphene, aqueous solution of graphene immediately after adding saturated $Ca(OH)_2$ solution).

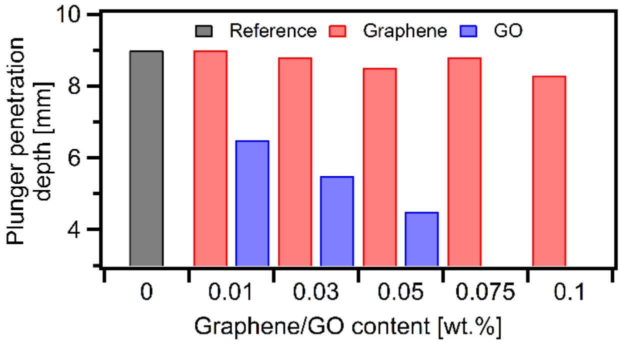

Figure S3. Results of consistency measurements for CEM II composites incorporating graphene and graphene oxide. Consistency measurements were performed using the plunger penetration method.

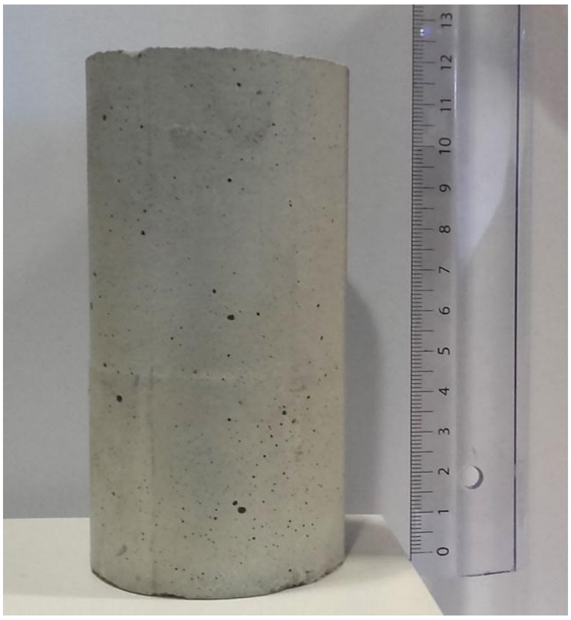

Figure S4. Cylindrical sample (with the diameter of 60 mm and the height of 120 mm) of cement mortar prepared for mechanical properties tests.

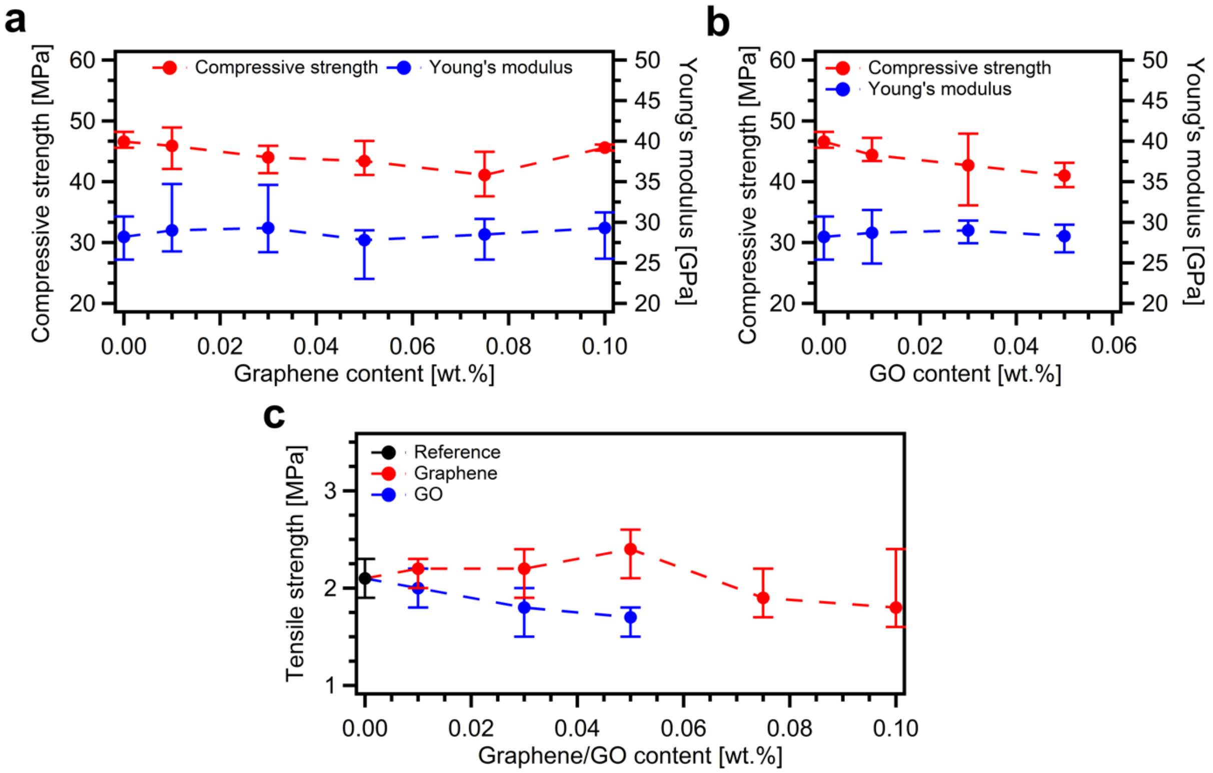

Figure S5. Mechanical properties of CEM II mortars at the age of 28 days. a) and b) Compressive strength and Young's modulus for composites incorporating a) graphene and b) graphene oxide. c) Tensile strength for specimens with graphene and GO.

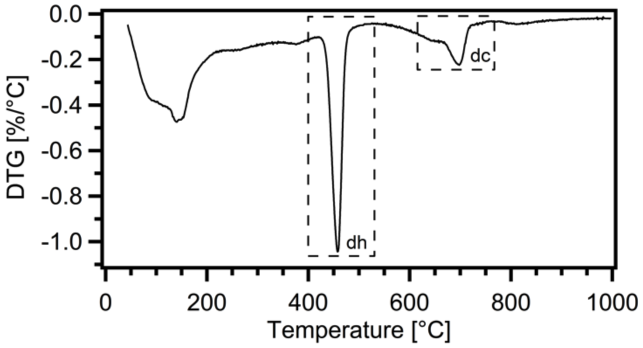

Figure S6. DTG curve for CI-R specimen. Dh represents the dehydroxylation region at the temperature range between 400°C and 530°C, while dc accounts for the decarbonation at the temperature range from 600°C to 750°C.

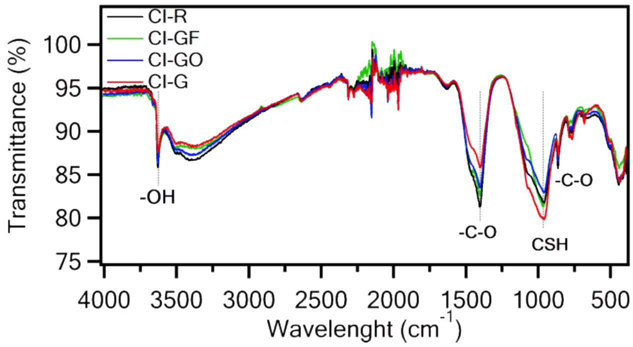

Figure S7. Full Fourier transform infrared spectroscopy (FT-IR) spectra for CI-R, CI-G-0.05, CI-GO-0.05, CI-GF-0.05 samples at the age of 28 days revealing the main peaks attributed to -OH of $Ca(OH)_2$, -C-O of $CaCO_3$ and Si-O bonds of C-S-H phase.

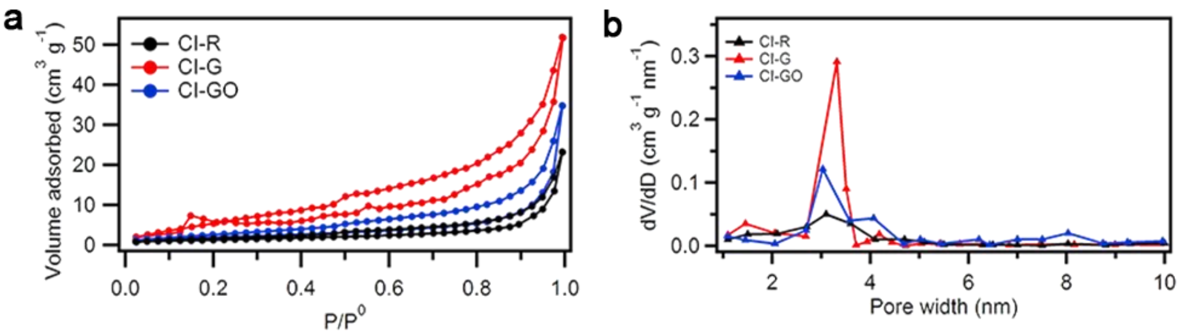

Figure S8. a) Nitrogen adsorption–desorption curves, and b) pore size distribution plots for CI-R, CI-G-0.05 and CI-GO-0.05 mesoporous composites.

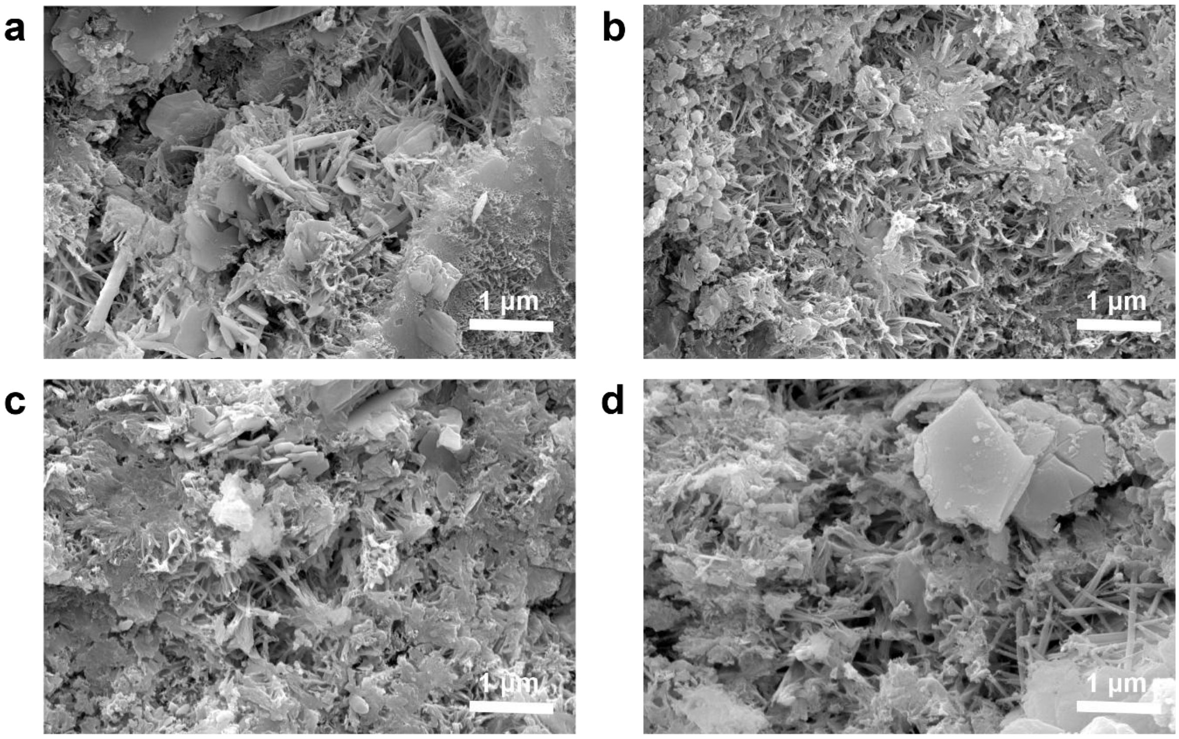

Figure S9. Morphological characterization of tested CEM I samples. SEM images for a) CI-R, b) CI-G-0.05, c) CI-GO-0.05, and d) CI-GF-0.05 samples at the age of 28 days showing the microstructure of hardened cement mortars with visible needle-like crystals of ettringite, plate-like crystals of calcium hydroxide and amorphous C-S-H phase.

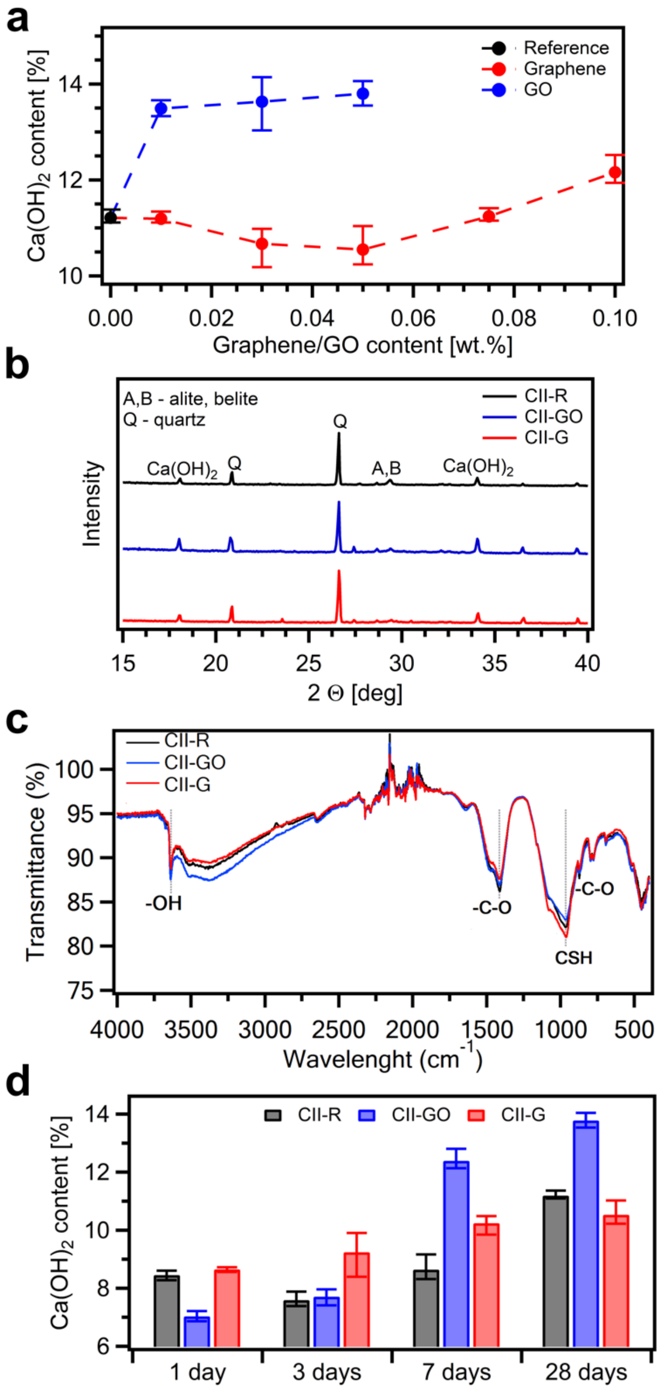

Figure S10. Structural characterization of CEM II composites. a) $Ca(OH)_2$ content at the age of 28 days in cement mortars incorporating graphene and graphene oxide. b) XRD patterns and c) FTIR spectra for CII-R, CII-GF-0.05, CII-GO-0.05 and CII-G-0.05 samples at the age of 28 days. d) $Ca(OH)_2$ content at the age of 1, 3, 7 and 28 days in CII-R, CII-GF-0.05, CII-GO-0.05 and CII-G-0.05 samples.

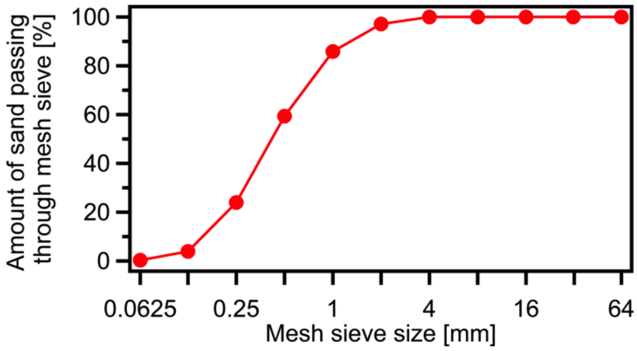

Figure S11. Results of sand sieve analysis. Analysis was performed with the use of the set of ten sieves with the mesh sieve size ranging from 31.5 mm to 0.063 mm.
